# Risks of Hemolysis in Glucose-6-Phosphate Dehydrogenase Deficient Infants Exposed to Chlorproguanil-Dapsone, Mefloquine and Sulfadoxine-Pyrimethamine as Part of Intermittent Presumptive Treatment of Malaria in Infants

**DOI:** 10.1371/journal.pone.0142414

**Published:** 2015-11-23

**Authors:** Eugenie Poirot, Eric Vittinghoff, Deus Ishengoma, Michael Alifrangis, Ilona Carneiro, Ramadhan Hashim, Vito Baraka, Jacklin Mosha, Samwel Gesase, Daniel Chandramohan, Roland Gosling

**Affiliations:** 1 Global Health Group, University of California San Francisco, San Francisco, California, United States of America; 2 Department of Epidemiology and Biostatistics, University of California San Francisco, San Francisco, California, United States of America; 3 Tanga Medical Research Centre, National Institute for Medical Research, Tanga, United Republic of Tanzania; 4 Centre for Medical Parasitology, Department of International Health, Immunology and Microbiology, University of Copenhagen, Copenhagen, Denmark; 5 Department of Infectious Diseases, National University Hospital (Rigshospitalet), Copenhagen, Denmark; 6 Department of Infectious and Tropical Diseases, London School of Hygiene and Tropical Medicine, London, United Kingdom; 7 Mwanza Interventions Trial Unit, National Institute for Medical Research, Mwanza, United Republic of Tanzania; 8 International Health Unit, Department of Epidemiology, University of Antwerp, Antwerp, Belgium; Quensland University of Technology, AUSTRALIA

## Abstract

**Background:**

Chlorproguanil-dapsone (CD) has been linked to hemolysis in symptomatic glucose-6-phosphate dehydrogenase deficient (G6PDd) children. Few studies have explored the effects of G6PD status on hemolysis in children treated with Intermittent Preventive Treatment in infants (IPTi) antimalarial regimens. We sought to examine the joint effects of G6PD status and IPTi antimalarial treatment on incidence of hemolysis in asymptomatic children treated with CD, sulfadoxine-pyrimethamine (SP), and mefloquine (MQ).

**Methods:**

A secondary analysis of data from a double-blind, placebo-controlled trial of IPTi was conducted. Hemoglobin (Hb) measurements were made at IPTi doses, regular follow-up and emergency visits. G6PD genotype was determined at 9 months looking for SNPs for the A- genotype at coding position 202. Multivariable linear and logistic regression models were used to examine hemolysis among children with valid G6PD genotyping results. Hemolysis was defined as the absolute change in Hb or as any post-dose Hb <8 g/dL. These outcomes were assessed using either a single follow-up Hb on day 7 after an IPTi dose or Hb obtained 1 to 14 or 28 days after each IPTi dose.

**Findings:**

Relative to placebo, CD reduced Hb by approximately 0.5 g/dL at day 7 and within 14 days of an IPTi dose, and by 0.2 g/dL within 28 days. Adjusted declines in the CD group were larger than in the MQ and SP groups. At day 7, homo-/hemizygous genotype was associated with higher odds of Hb <8 g/dL (adjusted odds ratio = 6.7, 95% CI 1.7 to 27.0) and greater absolute reductions in Hb (-0.6 g/dL, 95% CI -1.1 to 0.003). There was no evidence to suggest increased reductions in Hb among homo-/hemizygous children treated with CD compared to placebo, SP or MQ.

**Conclusions:**

While treatment with CD demonstrated greater reductions in Hb at 7 and 14 days after an IPTi dose compared to both SP and MQ, there was no evidence that G6PD deficiency exacerbated the adverse effects of CD, despite evidence for higher hemolysis risk among G6PDd infants.

## Introduction

Substantial progress has been made in malaria control over the last decade, with many malaria endemic countries now planning for malaria elimination [1]. The path to malaria elimination is multi-faceted, requiring the detection of clinical cases and targeting of asymptomatic infections where parasite reservoirs are likely to persist and perpetuate onward transmission [2]. Antimalarial drugs play a central role in this endeavor; however, certain antimalarial drugs that are key to many control and elimination strategies are unsafe among patients with glucose-6-phosphate dehydrogenase (G6PD) deficiency and can cause hemolysis.

G6PD deficiency is the most common X-linked enzyme deficiency in humans, affecting more than 400 million people worldwide [3]. Due to the X-linked nature of this deficiency, females can be homozygous or heterozygous while males can only be hemizygous for the gene. In consequence, partial inactivation of one female X chromosome or lyonization in somatic cells, result in varying enzyme activity among heterozygous females depending on the proportion of G6PD normal and G6PD deficient (G6PDd) red cells in their blood [4]. G6PD deficiency is relatively common in historically malaria endemic countries. This overlap is not a coincidence, as evidence suggests that G6PD deficiency arose through natural selection by malaria: the parasite appears to undergo adaptive changes in G6PDd cells to confer protection against malaria [5].

While variants of G6PD deficiency appear to provide partial protection against malaria [6–8] it can also cause hemolysis after exposure to certain triggers, such as the ingestion of certain foods (fava beans), infection (Hepatitis viruses A and B, cytomegalovirus, pneumonia, and typhoid fever) and exposure to oxidant drugs [3, 9–13]. Drug-induced G6PD deficiency-related hemolysis has been reported following therapy with a range of antimalarial drugs, including primaquine, methylene blue, and the sulphone drug dapsone [14]. Additionally, dapsone is used for a variety of indications [15], including in the treatment of leprosy, varied skin conditions, and more recently *Pneumocystis carinii* [16], especially in patients with HIV infection, but can also be used in combination with antimalarial drugs (pyrimethamine, proguanil and chlorproguanil) for malaria chemoprophylaxis and treatment [17].

In the late 1990s, chlorproguanil-dapsone (CD) was developed by a public-private partnership as a low-cost treatment for uncomplicated *Plasmodium falciparum* (*P*. *falciparum*) malaria in response to concerns of growing resistance to chloroquine and sulfadoxine-pyrimethamine (SP) in Africa [16, 18]. CD was later withdrawn from the market in 2008 following demonstration of post-treatment hemolytic anemia in G6PDd patients in two phase III trials [19, 20]. Prior to this, in 2004, Gosling *et al*. undertook a study to examine the protective efficacy and safety of three antimalarials–SP, mefloquine (MQ) and CD in an area of high SP resistance in northeast Tanzania in search of alternative regimens to SP for Intermittent Preventive Treatment for malaria in infants (IPTi) [21]. At the time, SP was used as first line treatment for uncomplicated malaria nationally in Tanzania. Findings from this study—the Kilimanjaro IPTi Drug Options Trial—showed that IPTi with the long acting drug MQ substantially reduced the incidence of clinical episodes of malaria in children, but neither SP nor CD showed signs of any protective efficacy against clinical malaria. High levels of SP resistance markers, owing to evidence of increasing selection for individual dihydrofolate reductase (*Pfdhfr*) and dihydropteroate synthetase (*Pfdhps*) mutations, offers an explanation for this lack of efficacy in episodes of clinical malaria [22]. Regarding the safety of these three antimalarial drugs, more deaths were observed in infants in the CD and MQ groups than in the SP or placebo groups. The study also noted that children in the CD group experienced greater declines in mean hemoglobin concentrations and had a higher risk of moderate anemia (hemoglobin <8 g/dL) 7 days after an IPTi dose. Children in the CD group also had a shorter time to first or only episode of moderate anemia than did those in the placebo or other drug groups [21]. Despite these patterns, none of the analyses specifically documented the effect of G6PD status on hemolysis and other adverse events, including hospitalization, blood transfusion, or death, in children treated with these three IPTi antimalarial regimens. To our knowledge, this analysis is the first to thoroughly examine the effects of G6PD deficiency in the context of IPTi in asymptomatic children under the age of one year.

To address this gap, using existing data from the Kilimanjaro IPTi Drug Options Trial, we explored differences in susceptibility to hemolysis and other adverse events among asymptomatic G6PDd infants treated with three antimalarial drugs in sub-Saharan Africa. Here, we examine the joint effects of G6PD status and IPTi antimalarial treatment on incidence of hemolysis. We hypothesized that incidence of hemolysis is increased among homo-/hemizygous infants treated with CD compared to placebo and other antimalarials, especially within 7 days following treatment. First, we contrast absolute changes in hemoglobin levels up to 7, 14 and 28 days after an IPTi dose by treatment. Then we estimate the impact of G6PD status on hemolysis among all infants who received active antimalarial treatment and assess for evidence of modification of the effects of IPTi treatment on hemolysis by G6PD status. Finally, we assess G6PD genotype effects on incidence of other adverse event episodes up to two years of age following IPTi.

## Methods

### Study design and study site

This is a secondary analysis of data from a clinical trial that took place between 2004 and 2008. The drug trial has been described in detail elsewhere [21]. The protocol is also available as supporting information (**S1 Protocol**). Briefly, the trial tested the protective efficacy and safety of three antimalarial regimens for IPTi. The trial was a randomized, double-blind placebo-controlled trial of SP, CD, and MQ, compared to placebo, conducted at neighboring moderate- and low-transmission sites in northeast Tanzania. Children aged 8–16 weeks who attended clinics for WHO’s Extended Program on Immunization (EPI) were eligible for inclusion. Enrolled children who met the inclusion criteria were randomly assigned to receive full treatment doses of SP, CD, MQ, or placebo, given alongside routine immunizations at approximately 2, 3, and 9 months of age. Blood samples were collected before children were given their first and third course of IPTi. Blood was also collected on filter paper bloodspots (Whatman 3MM) using the buffy coat. Hemoglobin measurements were made at IPTi doses, regular follow-up and emergency visits. A total of 800 infants had their hemoglobin concentration systematically measured on day 7 after IPTi drug administration, including the first 200 infants to receive the first course of IPTi and the second 200 infants to receive the third course at each site. Every child was followed up at home on days 2 and 3 following treatment. During these visits, health workers assessed for possible adverse events and checked for adherence to drug regimens. In addition, all children were followed up at 10, 18, and 24 months of age. Additional follow-up visits at 11 and 12 months of age were done on random samples (**S1 Fig**).

### G6PD (A-) genotyping

G6PD screening was conducted using samples collected at routine visits at 9 months, focusing on the G6PD deficiency allele 202A G6PD A-, the most common in sub-Saharan Africa. Whole blood samples were collected and centrifuged on the day of collection. Plasma, buffy coat and red cells were stored at -20°C locally and transferred to the central laboratory for G6PD testing at a later date. Filter paper bloodspots (Whatman 3MM) for each patient were prepared using the collected buffy coat. DNA extraction from the impregnated filter papers was done using the Chelex method as previously described [23]. The extracted DNA samples were genotyped using a simple high-throughput sequence-specific oligonucleotide probe (SSOP)-ELISA method described by Enevold *et al*. [24] to detect the most prominent single nucleotide polymorphisms (SNPs) in the G6PD genes (B, A and A-).

### Ethics

The clinical protocol of the original study was approved by the National Medical Research Coordination Committee of the National Institute for Medical Research of Tanzania (NIMR-MRCC) and by the London School of Hygiene and Tropical Medicine ethics committee and registered with ClinicalTrials.gov (identifier: NCT00158574).

### Study population

For this analysis, only children whose G6PD status could successfully be determined were included.

### Variables and definitions

Homozygous females and hemizygous males were considered G6PDd and analyzed together, because of small numbers. Heterozygous G6PD females were analyzed as a separate category.

The primary safety endpoint considered for this analysis was incidence of hemolysis. We used hemoglobin as a surrogate for hemolysis using two measures. First, hemolysis was defined as the absolute change in hemoglobin, from the day of the most recent IPTi dose. We also defined hemolysis as any post-dose hemoglobin measurement <8 g/dL (a measure of moderate anemia).

For each outcome definition, we evaluated hemolysis using two approaches. The first restricted the analysis to children who had their hemoglobin concentration systematically measured on day 7 after an IPTi dose. The second considered any follow-up hemoglobin measurements obtained from 1 to 14 or 28 days after each IPTi dose administration, provided it was obtained before the next IPTi dose. The time frames for follow-up hemoglobin measurements were chosen to reflect what is known about the duration of drug-induced hemolytic episodes. Typically, within 24–72 hours of drug dosing, clinically detectable hemolysis and jaundice become apparent, worsening until days 7–8 [3]. Studies have shown that hemoglobin concentrations after use of oxidant drugs are typically lowest on day 7 [19, 25, 26]. Once such drugs are discontinued, hemoglobin concentrations begin to recover after 8–10 days [3].

We also assessed treatment and genotype effects on incidence of adverse events (malaria, hospitalizations, blood transfusions, and death) throughout the study period.

Directed acyclic graphs were used to identify potential confounders of G6PD status. In contrast to many conventional epidemiologic risk factors, genotype is not affected by most risk factors for hemolysis or malaria. However, G6PD deficiency does vary by sex, a potential risk factor for study outcomes. Moreover, genotype varies by distance above sea level (and accordingly site), a risk factor for malaria if not hemolysis, probably due to the selective pressure of malaria endemicity on the source population. Accordingly, genotype effect estimates were adjusted for these potential confounders.

### Statistical analysis

All analyses were performed using STATA 13.1 (STATA Corporation, College Station, TX, USA).

Linear and logistic models were used for continuous and binary hemolysis outcomes measured only once for each infant. For repeated continuous and binary outcomes, mixed effects linear and logistic models with random intercepts were used. Analyses exploring G6PD genotype effects on hemolysis were restricted to children in the active arms, motivated by the hypothesis that adverse effects of G6PD deficiency would only be observed in infants receiving active treatment. Analyses were adjusted for sex and elevation, either directly or using inverse weighting by way of propensity scores for rare binary and failure time outcomes. Propensity scores, an alternative approach to standard adjustment for covariates, are useful when a binary or categorical exposure is common, but the binary or failure time outcome is rare, and when there are a large number of potential confounders that must be accounted for [27]. In analyses of changes in hemoglobin, we also adjusted for treatment assignment, dose number, weight and elevation. For each hemolysis outcome, we assessed genotype-treatment interactions.

Poisson models were used to estimate genotype effects on incidence of hemolysis and secondary adverse event outcomes, adjusting for treatment, gender, site, and elevation, with follow-up censored at 24 months of age or on the date of exit for children lost to follow-up due to migration, refusal, or exclusion. As in the main trial analysis, children were considered not at risk for malaria for 21 days after receipt of treatment with an antimalarial drug, not at risk for hemolysis for 28 days after a hemolytic episode [28], and not at risk for hospitalization for 7 days after hospital admission.

## Results

### Baseline characteristics

**Fig 1:**
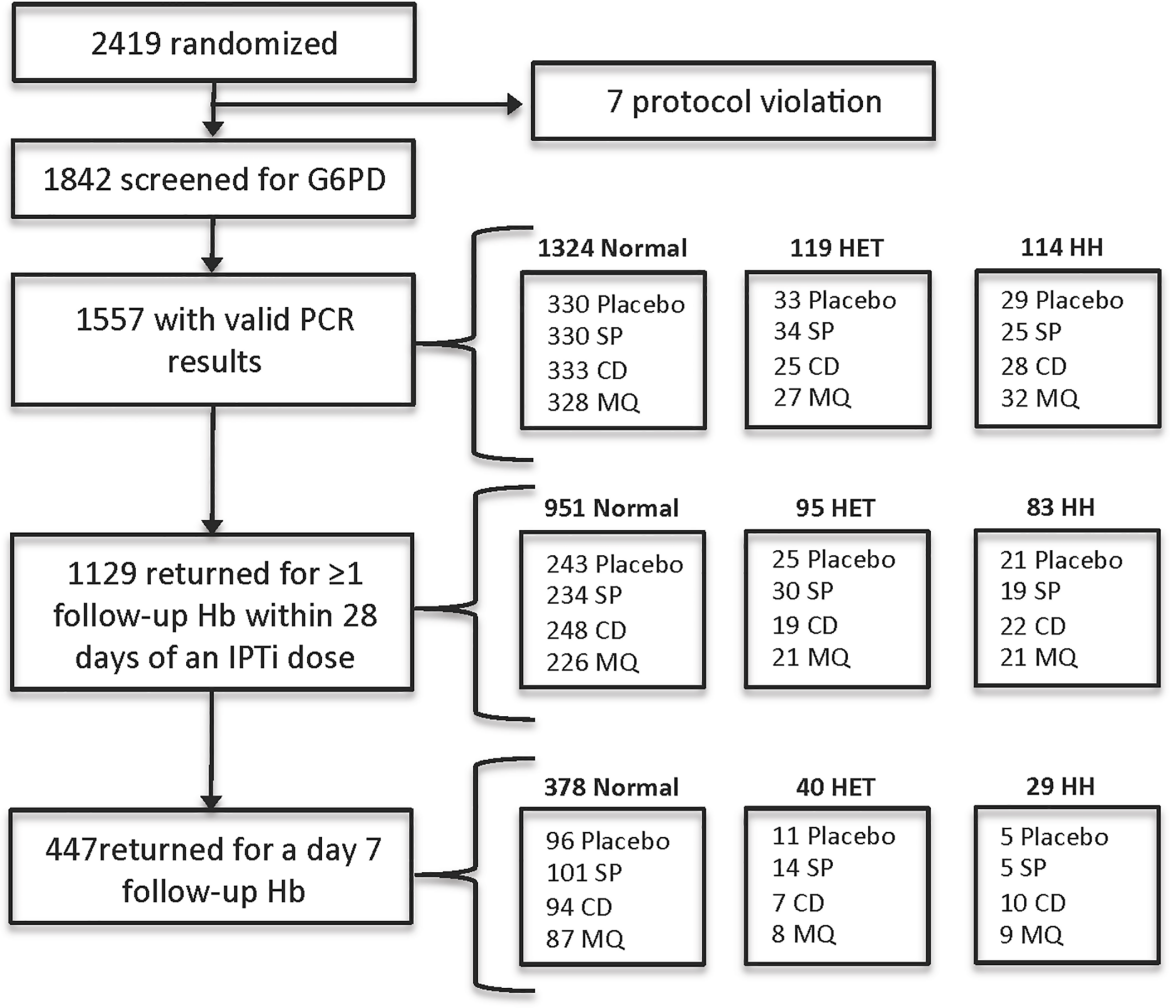
Trial profile for the entire cohort (both sites combined). SP = sulfadoxine-pyrimethamine. CD = chlorproguanil-dapsone. MQ = mefloquine. HET = heterozygous. HH = homo-/hemizygous.

A total of 2419 children were enrolled in the original trial, of whom 1842 (76%) were screened for G6PD deficiency. Among those screened, 1557/1842 (85%) were successfully genotyped and included in this analysis. G6PD results were not obtained for the remaining 285 patients (15%) either because samples were not received or DNA extraction was not successful. Among the genotyped children, 1129 (73%) returned for at least one follow-up hemoglobin measurement within 28 days of an IPTi dose. In addition, a randomly selected 447 (28%) returned for a day 7 follow-up hemoglobin measurement (**Fig 1**).

Demographic and clinical characteristics at enrollment among children with valid genotyping results were generally similar across genotypes, with a few exceptions (**Table 1**). Sex differences (p<0.001) are explained by the fact that the heterozygous genotype only exists in females. Children from the high transmission site (Korogwe) (p = 0.003) and those who lived at lower median elevations from sea level (p = 0.002) were more likely to be homo-/hemizygous. Homo-/hemizygous children also displayed lower median hemoglobin levels (p = 0.004) and slightly higher median weight values at enrollment (p = 0.005). Compared to children with successful G6PD genotyping results, included in this analysis, excluded children (those with no or invalid G6PD genotype, n = 855) were more likely to be male (p = 0.004), lived closer to the nearest clinic (p = 0.002), and displayed higher bednet coverage (p = 0.02) (**S1 Table**).

**Table 1:** Demographic and clinical data at enrollment among G6PD genotyped children with successful G6PD genotyping results.

Characteristic	Normal (n = 1324)	Heterozygous (n = 119)	Homo-/hemizygous (n = 114)	P-value
Age (weeks)	9.0[8.0–10.0]	9.0[8.0–10.0]	9.0[9.0–10.0]	0.3
Weight (kg)^δ^	5.5[5.1–6.0]	5.5[5.0–5.9]	5.7[5.2–6.2]	0.005 ^Λ^
Hemoglobin (g/dL)^λ^	10.7[9.9–11.5]	10.7[10.0–11.4]	10.2[9.3–11.3]	0.004^Λ^
Elevation from sea level (m)^§^	359[315–580]	355[311–565]	329[310–541]	0.002
Distance to nearest clinic (km)^Φ^	2.2[1.1–4.6]	2.2[1.0–4.9]	1.7[0.9–4.2]	0.2
Witnessed bednet coverage	1169(88.3)	104(87.4)	101(88.6)	1.0
Reported ITN coverage	724(54.7)	69(58.0)	67(58.8)	0.6
Rural residence^¶^	819(62.0)	78(65.6)	70(61.4)	0.7
Girls	630(47.6)	119(100.0)	40(35.1)	[-]^τ^
Korogwe	700(52.9)	65(54.6)	79(69.3)	0.003
Treatment				
Placebo	333(25.2)	33(27.7)	29(25.4)	0.8
SP	330(24.9)	34(28.6)	25(21.9)	
CD	333(25.2)	25(21.0)	28(24.6)	
MQ	328(24.8)	27(22.7)	32(28.1)	

Data are median [IQR] or n (%). SP = sulfadoxine-pyrimethamine. CD = chlorproguanil-dapsone. MQ = mefloquine.

^δ^Data missing for 3 patients in the normal group.

^λ^Data missing for 17 patients in the normal group, 1 patient in the homo-/hemizygous group, and 1 patient in the heterozygous group.

^§^Data missing for 2 observations in the normal group.

^Φ^Data missing for 2 patients in the normal group.

^¶^Data missing for 2 patients in the normal group.

^Λ^A parametric test was used after assessment of normality.

^τ^ The probability of a heterozygote being female is 1; the event is certain to occur and cannot be explained by chance.

### Treatment effects on changes in hemoglobin up to 28 days after an IPTi dose

In analyses adjusting for genotype, sex, dose number, site, and elevation, treatment with CD reduced hemoglobin levels by approximately 0.5 g/dL 7 days after an IPTi dose, by a similar amount within 14 days, and by 0.2 g/dL within 28 days, compared to placebo (**Table 2**). Adjusted declines in the CD group were also generally larger than in the MQ and SP groups, which did not differ from placebo.

**Table 2:** Adjusted treatment effects on changes in hemoglobin 7, 14, and 28 days after an IPTi dose.

Adjusted between-group differences (g/dL) [95%CI]									
	*7 days after an IPTi dose*			*14 days after an IPTi dose*			*28 days after an IPTi dose*		
Treatment	Estimate	P-value^a^	Overall p-value^b^	Estimate	P-value ^a^	Overall p-value^b^	Estimate	P-value^a^	Overall p-value^b^
Placebo	Ref		0.01	Ref		<0.001	Ref		0.07
SP	0.00 [-0.34 to 0.34]	1.0		0.06 [-0.22 to 0.34]	0.7		-0.07 [-0.28 to 0.13]	0.5	
MQ	0.10 [-0.26 to 0.45]	0.6		-0.03 [-0.31 to 0.26]	0.8		0.08 [-0.13 to 0.29]	0.5	
CD	-0.45 [-0.80 to -0.09]	0.01		-0.50 [-0.79 to -0.22]	0.001		-0.19 [-0.40 to 0.01]	0.07	

Analyses adjusted for genotype, dose number, sex, site, weight (kg) and elevation (m).

^**a**^P-values for adjusted treatment effects compared to chlorproguanil-dapsone (CD).

^**b**^P-values for overall adjusted treatment effects. SP = sulfadoxine-pyrimethamine. MQ = mefloquine.

### Genotype effects on hemolysis up to 28 days after an IPTi dose among children given active treatment

In analyses examining the impact of G6PD status on hemolysis among 329 children in the active treatment arms, adjusting for sex and elevation using inverse weighting, we found a strong association of homo-/hemizygous genotype with hemoglobin <8 g/dL 7 days after IPTi treatment (adjusted odds ratio = 6.7, 95% CI 1.7 to 27.0, p = 0.01) (**Table 3**). This was an uncommon outcome, with only 6 events (2%) in the normal group (n = 281), 4 events (17%) in the homo-/hemizygous group (n = 24), and no events in the heterozygous group (n = 29) (p = 0.01). On day 7, there was also borderline evidence of a greater average decline in hemoglobin among homo-/hemizygous children compared to normal children (-0.6 g/dL, 95% CI -1.1 to 0.003, p = 0.05). In contrast, we found little or no evidence for a genotype effect on either outcome within 14 or 28 days of an IPTi dose.

**Table 3:** Adjusted genotype effects on hemolysis 7, 14, and 28 days after an IPTi dose among treated infants.

	**Adjusted between-group differences (g/dL) [95% CI]**								
	*7 days after an IPTi dose*			*14 days after an IPTi dose*			*28 days after an IPTi dose*		
**Hemolysis endpoint**	**N**	**Estimate**	**P-value**	**N[Nobs]**	**Estimate**	**P-value**	**N[Nobs]**	**Estimate**	**P-value**
**Absolute declines** ^a^									
Normal	281	Ref		409[450]	Ref		706[913]	Ref	
Heterozygous	29	-0.08 [-0.6 to 0.5]	0.8	39[43]	-0.09 [-0.6 to 0.4]	0.7	70[87]	-0.2 [-0.5 to 0.2]	0.3
Homo-/hemizygous	24	-0.6 [-1.1 to 0.003]	0.05	34[39]	-0.4 [-0.8 to 0.1]	0.1	62[79]	-0.3 [-0.6 to 0.1]	0.1
	**Adjusted odds ratio [95% CI]**								
	*7 days after an IPTi dose*			*14 days after an IPTi dose*			*28 days after an IPTi dose*		
	**N**	**Estimate**	**P-value**	**N[Nobs]**	**Estimate**	**P-value**	**N[Nobs]**	**Estimate**	**P-value**
**Declines to less than 8 g/dL** ^b^									
Normal	281	Ref		451[530]	Ref		769[1136]	Ref	
Heterozygous^c^	29	-	-	40[49]	0.9 [0.2 to 3.7]	0.8	74[101]	0.6 [0.2 to 2.1]	0.5
Homo-/hemizygous	24	6.7 [1.7 to 27.0]	0.01	35[41]	2.9 [1.03 to 8.4]	0.04	66[93]	1.3 [0.5 to 3.2]	0.6

Analyses were restricted to children in the active arms on the assumption that adverse effects of G6PD deficiency are only observed in infants receiving active treatment.

^a^Adjusted for intervention arm, dose number, sex, site, weight (kg) and elevation (m).

^b^Adjusted for sex and elevation (m) using inverse probability to treatment weights.

^c^All observations were dropped due to no events. N[Nobs] = number of unique infants[number of observations].

### Effect modification by G6PD status on incidence of hemolysis up to 28 days after an IPTi dose

In analyses stratified by genotype and adjusting for dose number, sex, site, weight and elevation, treatment with CD reduced hemoglobin by nominally larger amounts in the homo-/hemizygous group (range: 0.49–1.31 g/dL) than among normal genotype children (range: 0.18–0.48 g/dL) (**Table 4**), but there was no persuasive evidence for treatment-genotype interaction in any of these analyses (p>0.05, test for interaction of CD vs. placebo and homo-/hemizygous vs. normal). Due to few events, we were unable to explore effect modification by G6PD status on hemogobin <8 g/dL at day 7 or within 14 or 28 days of an IPTi dose.

**Table 4:** Adjusted treatment effects on changes in hemoglobin 7, 14, and 28 days after an IPTi dose, by G6PD genotype.

Adjusted between-group differences (g/dL) [95%CI]										
		*7 days after an IPTi dose*			*14 days after an IPTi dose*			*28 days after an IPTi dose*		
Genotype	Treatment	Estimate	P-value^a^	P-value, test for interaction^b^	Estimate	P-value^a^	P-value, test for interaction^b^	Estimate	P-value^a^	P-value, test for interaction^b^
**Normal**										
	Placebo	Ref		0.9(0.8)	Ref		0.5(0.2)	Ref		0.7(0.4)
	SP	-0.03 [-0.40 to 0.35]	0.9		0.09 [-0.22 to 0.40]	0.6		-0.03 [-0.25 to 0.20]	0.8	
	MQ	0.02 [-0.37 to 0.41]	0.9		-0.08 [-0.39 to 0.23]	0.6		0.07 [-0.16 to 0.30]	0.5	
	CD	-0.44 [-0.82 to -0.06]	0.02		-0.48 [-0.79 to -0.18]	0.002		-0.18 [-0.40 to 0.05]	0.1	
**Heterozygous**										
	Placebo	Ref			Ref			Ref		
	SP	0.38 [-0.68 to 1.44]	0.5		0.39 [-0.49 to 1.27]	0.4		-0.05 [-0.70 to 0.60]	0.9	
	MQ	0.48 [-0.74 to 1.70]	0.4		0.70 [-0.34 to 1.74]	0.2		0.07 [-0.66 to 0.79]	0.9	
	CD	-0.40 [-1.68 to 0.88]	0.5		-0.19 [-1.20 to 0.83]	0.7		-0.12 [-0.85 to 0.60]	0.7	
**Homo-/hemizygous**										
	Placebo	Ref			Ref			Ref		
	SP	-0.40 [-2.07 to 1.27]	0.6		-0.89 [-2.07 to 0.29]	0.1		-0.69 [-1.51 to 0.13]	0.1	
	MQ	0.41 [-1.06 to 1.87]	0.6		-0.34 [-1.51 to 0.83]	0.6		0.15 [-0.65 to 0.95]	0.7	
	CD	-0.65 [-2.10 to 0.80]	0.4		-1.31 [-2.43 to -0.19]	0.02		-0.49 [-1.25 to 0.28]	0.2	

Analyses adjusted for dose number, sex, site, weight (kg) and elevation (m).

^**a**^P-values for adjusted treatment effects by G6PD genotype compared to chlorproguanil-dapsone (CD).

^b^P-values for overall interaction in stratified analyses (p-values for interaction of CD vs. placebo and homo-hemi vs. normal). MQ = mefloquine. SP = sulfadoxine-pyrimethamine.

### Genotype effects on adverse event episodes up to 2 years of age

During a median follow-up time of 21.8 months, 314 children experienced at least one episode of clinical malaria, for a total of 707 episodes, including 598 in the normal genotype group, 67 among heterozygous children, and 42 among the homo-/hemizygous group. There were also a total of 954 hospitalizations: 362 participants had one hospitalization, 155 had two, and 85 were hospitalized three or more times. There was no evidence for differences in malaria incidence or hospitalizations by G6PD genotype. Thirty-four children received blood transfusions, including three who required more than one transfusion, for a total of 38 events. Seven children died, including two in the homo-/hemizygous group (one in the CD arm); the remaining deaths were among G6PD normal children. These outcomes were too uncommon for meaningful statistical analysis (**S2 Table**).

## Discussion

Our analysis of the effects of antimalarial regimens and G6PD status on hematologic parameters in asymptomatic children treated with IPTi showed that treatment with CD caused reductions in hemoglobin at 7 and 14 days after an IPTi dose, relative to placebo, SP, and MQ. We also found evidence for higher hemolysis risk among G6PDd infants. Although we found no clear evidence that G6PD deficiency exacerbated the adverse effects of CD, the study was not powered to detect an interaction between these two factors.

The transient but statistically significant decrease in hemoglobin after initiation of antimalarial treatment is consistent with previous studies examining the hematologic variations in pediatric uncomplicated *P*. *falciparum* malaria and corresponding trends during post-treatment follow-up [29]. Furthermore, the hemolytic potential of CD in this context has been widely documented and clinically important decreases in hemoglobin or related adverse events requiring medical intervention post-treatment have been noted [19, 20, 30, 31]. Our analysis also revealed a strong association of homo-/hemizygous G6PD genotype with post-dose incidence of anemia (hemoglobin <8 g/dL) 7 days after IPTi treatment. Homo-/hemizygous infants also displayed borderline significant evidence of greater absolute declines in hemoglobin compared with normal infants at day 7. This is consistent with expectations and the clinical-hematological picture of drug-induced acute hemolytic anemia in G6PDd patients [16, 32, 33].

Drug induced hemolysis is self-limiting in individuals with G6PD deficiency [34]. In this study we show that hemoglobin levels are temporarily reduced, with hemoglobin declines appearing less pronounced within 28 days. Research has shown that the mechanism for this is explained through the destruction of older red blood cells during drug exposure that are the most enzyme deficient while young erythrocytes with nearly normal levels of G6PD are resistant to destruction [34, 35].

Some limitations to our analysis should be noted. As previously described, many analyses were underpowered, in particular for treatment-genotype interactions, due to the low frequency of homo-/hemizygous and heterozygous genotypes. Assessing the hematological effects of antimalarials is complicated, especially when the risk of adverse reactions is confined to a relatively small subgroup of patients—those with G6PD deficiency—and demonstrating a statistically significant trend is unlikely. Further, we conducted multiple comparisons, potentially inflating the overall type-I error rate. Additionally, analyses were restricted to children who survived and remained in follow-up to 9 months of age, and had valid G6PD results, with the potential to induce selection bias and limit the generalizability of our findings. However, baseline characteristics of the children with valid G6PD results were generally similar to those who were excluded from the analysis. An additional limitation is that G6PD deficiency was determined by screening human DNA for a single SNP in the G6PD gene (G202A), although mutations other than G6PD A- could have affected hemolysis risk and explain part of the observed results. However, this is unlikely to be important, because G6PD A- accounts for 90% of G6PD deficiency in Africa [3].

These limitations render it challenging to find true subgroup effects, partially explaining why there is currently limited data concerning the hemolytic risk associated with diverse antimalarial drug regimens across G6PD genotypes in most populations. This analysis was underpowered to detect interaction; nonetheless, the direction of trends is consistent with expectations that IPTi with CD may produce greater reductions in hemoglobin among homo-/hemizygous children compared to other antimalarials. CD was withdrawn from the market in 2008 due to post-licensure hemolytic toxicity in patients with G6PD deficiency; however, dapsone is still used in the prevention and treatment of a variety of diseases [15–17, 36] and can precipitate life-threatening hemolysis in individuals with G6PD deficiency. In addition, two 8-aminoquinoline drugs with similar safety profiles, primaquine and tafenoquine, are attracting much interest as chemotherapeutic and prophylactic antimalarial agents against the liver stages of *P*. *vivax* and *P*. *falciparum*. Both drugs also cause oxidant hemolysis in individuals with G6PD deficiency, and as malaria control programs begin to consider the use of primaquine or tafenoquine (if approved and licensed), identifying subgroups for which antimalarial treatment may be harmful or likely to have the largest benefit will become increasingly important. While this may prove challenging given the rarity of G6PD deficiency, even if fixed by design, pooled analyses with larger samples of G6PDd individuals could offer new insights. Interaction analyses can enable us to substantiate clinically important differences in hematological effects for oxidative antimalarial treatments across G6PD genotypes, and approaches to examine such trends in this important patient population are needed.

## Conclusion

This re-analysis of data from the Kilimanjaro IPTi Drug Options Trial among nearly 1600 children from northeastern Tanzania showed that treatment with CD as well as G6PD deficiency were associated with declines in hemoglobin and increased risk of moderate anemia. However, despite a fairly large sample, the study was underpowered to determine whether the adverse effects of CD are exacerbated among infants with G6PD deficiency, primarily because these deficient G6PD genotypes were uncommon. Combining data from a series of similar, well-conducted primary studies could offer a way to answer this question and optimize the targeting of treatment, which is increasing in importance as systematic treatment with drugs with hemolytic potential, such as the 8-aminoquinolines, primaquine and tafenoquine, becomes more widespread.

## Supporting Information

S1 Fig Trial timeline for participants of the IPTi trial in Tanzania. (TIFF)

S1 Protocol Trial Protocol. (DOC)

S1 Table Demographic and clinical data at enrollment among children from entire cohort. (DOCX)

S2 Table Comparison of secondary outcomes of IPTi according to G6PD genotype using multivariable Poisson regression. (DOCX)
