# Supplementary figures and images for: Risks of Hemolysis in Glucose-6-Phosphate Dehydrogenase Deficient Infants Exposed to Chlorproguanil-Dapsone, Mefloquine and Sulfadoxine-Pyrimethamine as Part of Intermittent Presumptive Treatment of Malaria in Infants
Source: PLoS One. 2015 Nov 23;10(11):e0142414. doi: 10.1371/journal.pone.0142414 (PMC4658078; doi:10.1371/journal.pone.0142414)

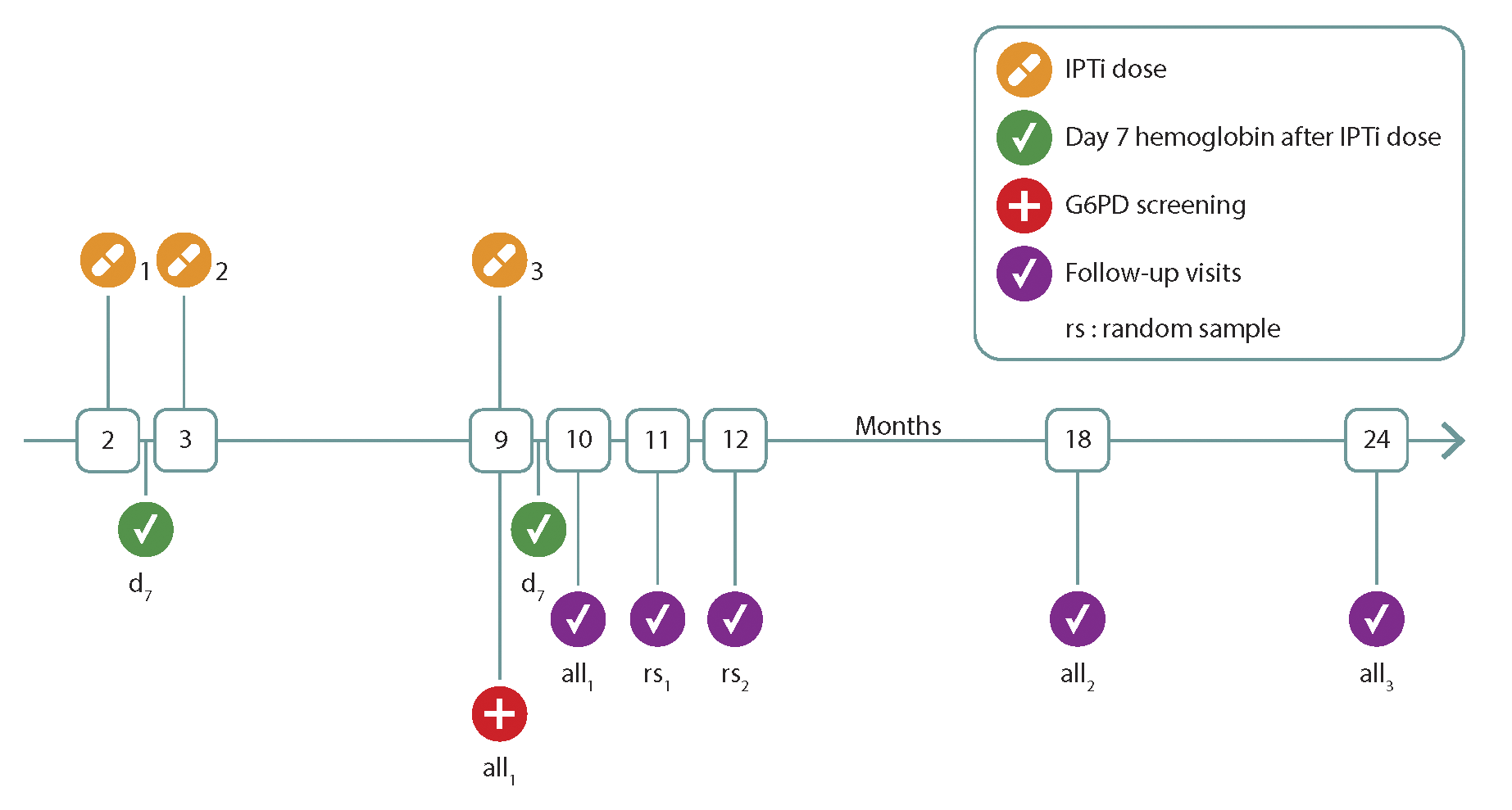

Supplement: S1 Fig — (TIFF) [file pone.0142414.s001.tiff]
