# Supplementary material for: Risks of Hemolysis in Glucose-6-Phosphate Dehydrogenase Deficient Infants Exposed to Chlorproguanil-Dapsone, Mefloquine and Sulfadoxine-Pyrimethamine as Part of Intermittent Presumptive Treatment of Malaria in Infants
Source: PLoS One. 2015 Nov 23;10(11):e0142414. doi: 10.1371/journal.pone.0142414 (PMC4658078; doi:10.1371/journal.pone.0142414)
Supplement: S1 Table — (DOCX) [file pone.0142414.s003.docx]

| Characteristic | Children with G6PD PCR results (n = 1557) | Children without PCR results (n = 855) | P-value* |
| --- | --- | --- | --- |
| Age (weeks) | 9.0[8.0-10.0] | 9.0[8.0-10.0] | 0.7 |
| Weight (kg)^δ^ | 5.5[5.1-6.0] | 5.5[5.2-6.1] | 0.6 |
| Hemoglobin (g/dL)^λ^ | 10.6[9.9-11.4] | 10.7[10.0-11.5] | 0.4^Λ^ |
| Elevation from sea level (m)^§^ | 354[314-573] | 366[315-580] | 0.2 |
| Distance to nearest clinic (km)^Φ^ | 2.1[1.1-4.6] | 1.8[1.0-3.8] | 0.002 |
| Witnessed bednet coverage | 1374(88.3) | 781(91.4) | 0.02 |
| Reported ITN coverage | 860(55.2) | 486(56.8) | 0.4 |
| Rural residence^¶^ | 967(62.1) | 498(58.3) | 0.08 |
| Girls | 789(50.7) | 381(44.6) | 0.004 |
| Korogwe | 844(54.2) | 432(50.5) | 0.08 |
| Treatment |  |  |  |
| Placebo | 395(25.4) | 209(24.4) | 1.0 |
| SP | 389(25.0) | 213(24.9) |  |
| CD | 386(24.8) | 216(25.3) |  |
| MQ | 387(24.9) | 217(25.4) |  |

Data are median [IQR] or n (%). SP = sulfadoxine-pyrimethamine. CD = chlorproguanil-dapsone. MQ = mefloquine. ^δ^Data missing for 2 patients with PCR results and 1 patient without PCR results; ^λ^Data missing from 19 patients with PCR results and 11 patients without PCR results ; ^§^ Data missing for 2 patients with PCR results and 7 patients without PCR results; ^Φ^Data missing for 2 patient with PCR results and 7 patients without PCR results. ^¶^Data missing from 2 patients with PCR results and 4 without PCR results. ^Λ^A parametric test was used after assessment of normality.
