# Supplementary material for: Risks of Hemolysis in Glucose-6-Phosphate Dehydrogenase Deficient Infants Exposed to Chlorproguanil-Dapsone, Mefloquine and Sulfadoxine-Pyrimethamine as Part of Intermittent Presumptive Treatment of Malaria in Infants
Source: PLoS One. 2015 Nov 23;10(11):e0142414. doi: 10.1371/journal.pone.0142414 (PMC4658078; doi:10.1371/journal.pone.0142414)
Supplement: S2 Table — (DOCX) [file pone.0142414.s004.docx]

|  | Number of events | PYAR | Unadjusted Incidence per 1000 infant-months [95% CI] | Marginal incidence per 1000 infant-months [95% CI] | Conditional  IRR | p-value |
| --- | --- | --- | --- | --- | --- | --- |
| **All episodes of malaria** |  |  |  |  |  |  |
| Normal | 598 | 27.8 | 21.5[19.9-23.4] | 23.6[20.5-26.7] | Ref |  |
| Heterozygous | 67 | 2.5 | 26.5[20.8-33.6] | 31.9[18.2-45.7] | 1.4[0.9-2.1] | 0.2 |
| Homo-/hemizygous | 42 | 2.4 | 17.6[13.0-23.8] | 14.6[6.6-22.5] | 0.6[0.4-1.1] | 0.09 |
| **All-cause hospital admissions** |  |  |  |  |  |  |
| Normal | 793 | 27.8 | 28.5[26.6-30.6] | 28.7[26.3-31.1] | Ref |  |
| Heterozygous | 77 | 2.5 | 30.3[24.2-37.9] | 35.4[25.9-44.9] | 1.2[0.9-1.6] | 0.2 |
| Homo-/hemizygous | 84 | 2.4 | 35.2[28.4-43.6] | 33.7[24.5-42.8] | 1.2[0.9-1.6] | 0.3 |
| **Blood transfusions** |  |  |  |  |  |  |
| Normal | 32 | 27.8 | 1.2[0.8-1.6] | 1.2[0.8-1.7] | Ref |  |
| Heterozygous | 2 | 2.5 | 0.8[0.2-3.1] | 0.9[-0.4-2.3] | 0.7[0.2-3.4] | 0.7 |
| Homo-/hemizygous | 4 | 2.4 | 1.7[0.6-4.5] | 1.4[-0.2-3.0] | 1.1[0.3-3.7] | 0.9 |
| **All-cause deaths** |  |  |  |  |  |  |
| Normal | 5 | 27.8 | 0.2[0.07-0.4] | 0.2[0.02-0.4] | Ref |  |
| Heterozygous | 0 | 2.5 | 0 [-] | 0 [-] | 0 [-] | [-] |
| Homo-/hemizygous | 2 | 2.4 | 0.8[0.2-3.3] | 0.7[-0.3-1.6] | 3.4[0.6-19.3] | 0.2 |

Adjusted for intervention, sex, site and elevation. PYAR =person-year at risk per 1000. IRR = incidence rate ratio
